# Chemical and Sensory Characterization of Cabernet Sauvignon Wines from the Chinese Loess Plateau Region

**DOI:** 10.3390/molecules24061122

**Published:** 2019-03-21

**Authors:** Ke Tang, Yan-Ru Xi, Yue Ma, Hui-Ning Zhang, Yan Xu

**Affiliations:** 1 Key Laboratory of Industrial Biotechnology of Ministry of Education, Jiangnan University, 1800 Lihu Ave, Wuxi 214122, China; tandy81@jiangnan.edu.cn (K.T.); xiyanru@163.com (Y.-R.X.); 7160201007@vip.jiangnan.edu.cn (Y.M.); 2 State Key Laboratory of Food Science and Technology, Jiangnan University, 1800 Lihu Ave, Wuxi 214122, China; 3 Chateau Rongzi Company Ltd., Linfen 042100, China; huiningz@126.com

**Keywords:** Loess Plateau, Cabernet Sauvignon, aroma, GC–O, sensory profile

## Abstract

In this study, the aroma profiles of Cabernet Sauvignon wines from a new grape growing region, Loess Plateau, China, were established by gas chromatography–olfactometry, gas chromatography–mass spectrometry and sensory analysis. The sensory profiles of wines form five different young vineyards in the Loess Plateau region were obtained by descriptive analysis. Blackcurrant (*p* < 0.01), pear and dried plum (*p* < 0.05), mushroom, smoked and green pepper (*p* < 0.1) had significant differences on the five vineyards. A total of 76 odor-active aroma compounds were identified in the wines, and 45 volatile compounds were selected as those having the greatest impact on the aroma components and these were quantitated by five different methods. In addition, the correlation model of the Loess Plateau region’s sensory characteristics and aroma compounds was established by partial least squares regression (PLSR) to determine the influence of various aroma active substances on aroma attributes.

## 1. Introduction

Wine aroma, which mainly consists of alcohols, ethers, fatty acids, aldehydes, ketones and terpenes [1], is a key aspect of wine quality [2], and is also one of the most influential properties for consumers when buying wine [3]. The aroma is the result of the combined contribution of various volatile compounds and can rarely be attributed to a single, specific component [4]. The volatile compounds have a wide concentration range varying from hundreds of mg/L to the μg/L or ng/L level [5,6], but their contribution to the overall wine aroma is not proportional to their content [7].

Why are there such big differences in the aroma? For young wines, the composition and content of the aroma compounds largely depend on the vineyards, also referred to as the “terroir” [8]. The term “terroir” in French includes such characteristics as soil type, climate (sunlight, temperature and rainfall) and topography [9]. There has been much research demonstrating regional wines typicality. Some authors in Brazil have found that the aroma profiles of Cabernet Sauvignon wines from five vineyards of Santa Catarina State, Brazil, were established using gas chromatography–olfactometry (GC–O). Two wines have totally different aroma characteristics in sensory analysis, one with vegetative characteristics (Saõ Joaquim vineyard) and one with red fruits and jam aromas (Bom Retiro vineyard) [10]. Robinson et al. explored the relationship between the sensory characteristics and wine composition of Cabernet Sauvignon wines in relation to Australian geographical indications [11]. Another study compared the flavor characteristics of Vidal icewines from China and Canada. The Vidal icewines from China generally had more nut and honey aromas, while the Canadian Vidal icewines had more caramel and rose aromas [12].

As a rapidly developing wine-making country, China has formed a serious of special grape-growing regions, and has a large consumer population. These grape-growing regions are distributed from east to west, across China with a distance of over 3000 km [13]. In 2017, China consumed 1.79 million kiloliters of wine, which is the fifth highest amount in the world, and produced 1.08 million kiloliters of wine, which is the seventh highest amount in the world (International Organization of Vine and Wine, OIV). The Loess Plateau region is located in the middle reaches of the Chinese Yellow River. As it is easy to form a microclimate in unique plateau areas, this has a great influence on the quality of the grape and the wine. The Loess Plateau region has been recognized as one of the best areas for grape growing in China. Cabernet Sauvignon is the main variety in this region. However, limited information is available on the sensory and chemical composition of the wines there [14].

Chemical and sensory analyses are powerful tools that permit one to know the flavor of the wines. As is well known, gas chromatography has been extensively applied for measuring volatile components in the aroma of the wine [15]. In addition, GC–O can be considered a useful tool for the identification of the odor-active compounds in wine and a combination between sensory and instrumental analyses [16,17,18]. Many methods such as dilution methods and time-intensity methods have been applied to determine the olfactometric indices [19]. While sensory analysis can involve the odorant’s synergistic effect, GC–O analysis cannot. It includes the description of the qualitative and quantitative sensory components of wine by a trained panel. Additionally, the compounds in GC–O analysis were significantly separated and concentrated before sniffing, thus differing to the authentic wines. Descriptive analysis (DA) has proven particularly useful in studies of sensory evaluation in wines and can offer a complete description for all the sensory properties of wine [20].

The aim of this study was to identify the aroma characteristics of Cabernet Sauvignon wines made from five young vineyards of Loess Plateau, which is a new grape growing region of China. The Cabernet Sauvignon wines were analyzed for their olfactory profile and volatile composition by sensory analysis, GC–O and GC–MS analysis. Furthermore, all these sensory attributes were correlated in a partial least squares regression (PLSR) to instrumental-chemical measurements of volatiles, to study the sensory and chemical compounds associated with different wines.

## 2. Results and Discussion

### 2.1. Sensory Analyses

**Figure 1:**
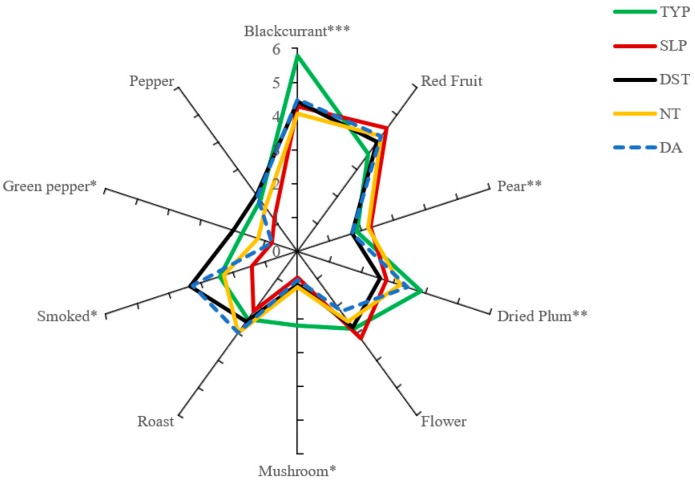
Aroma spider graphs of the sensory characteristics of Cabernet Sauvignon wine, obtained using 12 panelists with wines analyzed in triplicate. Asterisks indicate significance at * *p* < 0.1, ** *p* < 0.05, *** *p* < 0.01. SLP, TYP, NT, DA and DST are the abbreviations for five young vineyards in the Loess Plateau region.

The aroma profiles of five different Cabernet Sauvignon wines were described by the sensory panel. A total of 10 aroma descriptors were discussed and confirmed. They were blackcurrant, red fruit, pear, dried plum, flower, mushroom, roast, smoked, green pepper, and pepper. The results of the DA analysis are shown in Figure 1. As can be seen, blackcurrant is the descriptor in which all the samples scored high. In contrast, there are other descriptors, such as green pepper, pepper, pear and mushroom, for which the five samples reached low scores. The terms smoked, roast, floral, and dried plum reached high scores in some of the samples.

Several attributes (red fruit, flower, roast, and pepper) did not show significant *p* values among the five vineyards. These attributes suggest that these sensory descriptors are the common flavor notes of Cabernet Sauvignon in Loess Plateau, and that there are no significant differences between different vineyards. On the contrary, blackcurrant had a highly significant influence on the five vineyards with a 99% confidence level (*p* < 0.01) that means blackcurrant made a very different contribution among these vineyards. The Cabernet Sauvignon wines also showed significant differences in the aroma descriptors pear and dried plum (*p* < 0.05) and mushroom, smoked and green pepper (*p* < 0.1).

Wines from TYP were associated with more black berry and green notes and less red berry notes, whilst wines from SLP were described as more sweet and ripe and less herbaceous than other vineyards. The Cabernet Sauvignon wines from NT were perceived as closer to the DST Cabernet Sauvignon wines in aroma profile, but somewhat different from them. Compared to other wines in the present study, DST Cabernet Sauvignon wines were described as having less intense fruity, flower and vegetal notes, but higher smoked, roast and pungent notes. The DA Cabernet Sauvignon wines are evaluated had a more smoked and roast aroma.

### 2.2. Chemical Analysis

#### Olfactometric Data Selection of Cabernet Sauvignon Wines

In this step, three judges who are experienced in GC–O analysis carried out an olfactory evaluation using a three-point scale on the five wines. The data included the chromatographic retention times of odor detections, and odor descriptions and intensities (Table 1). Following this procedure, a total of 76 aroma compounds were identified in the five Cabernet Sauvignon wines in this study. Among them, the highest Osme value of the aroma compounds was determined for β-damascenone and 2,3-butanedione. β-damascenone exhibited honey and floral odors and 2,3-butanedione presented a butter aroma. Both of these two compounds were previously reported as important odorants in red wines [19]. Four further compounds with Osme values above 2.5 in five wines were identified as 3-Methyl-1-butanol (malt), Ethyl caprylate (orange), 3-(Methylthio)-Propionaldehyde (cooked potato) and Octanoic Acid (cheese). Seven of the 76 odors detected by the judges, were not identified by GC–MS, probably because their concentrations were below the method detection limit. Thirty active odors were common for every wine.

Volatile thiols are a group of aroma compounds with a significance to wine aroma, particularly Sauvignon Blanc and Cabernet Sauvignon wines, that has been widely studied [1]. Some of the most important of these are 4-Mercapto-4-methylpentan-2-one (4MMP), 3-(Methylthio)-propionaldehyde, 4-Mercapto-4-methyl-2-pentanol (4MMPol), 3-mercaptohexan-1-ol (3MH) and 3-Mercaptohexyl acetate (3MHA), which have aromas described as blackcurrant, grapefruit, passionfruit and boxwood [21].

A total of 45 aroma compounds with high Osme values (≥1.5) were further quantified. Five different methods were involved in aroma quantitation (Table 2). These aroma compounds had more influence on Cabernet Sauvignon wines from the Chinese Loess Plateau region. Currently, no one analytical method can quantify all of the volatile compounds accurately because of the different properties of aroma components. Therefore, five different methods were used for the quantification of these 45 compounds.

Table 3 presents the concentration of the 45 aroma active compounds. Data in the table have been arranged into ten chemical families (esters, alcohols, aldehyde, ketones, aromatic compounds, terpenes, sulfide, furan, acids, and pyrazines). Esters and alcohols were the major group of volatile compounds in all the wines, followed by terpenes.

ANOVA was used to determine differences between the regional volatile compounds’ concentration analysis, using Duncan with a 95% confidence level (*p* < 0.05). If the contents of two vineyards marked the same letter in the same row, it suggested that the contents of this compound had no significant difference in the two vineyards. On the other hand, if the contents of two vineyards marked the same letter, it suggested that the contents of this compound had a significant difference with a 95% confidence level (*p* < 0.05). There were four aroma components of Cabernet Sauvignon wines that had no significant difference in the five vineyards. They were (e)-3-hexen-1-ol, 1-octen-3-one, (e)-nerolidol and 2-methoxy-3-isobutyl pyrazine.

### 2.3. Multivariate Statistical Analysis

**Figure 2:**
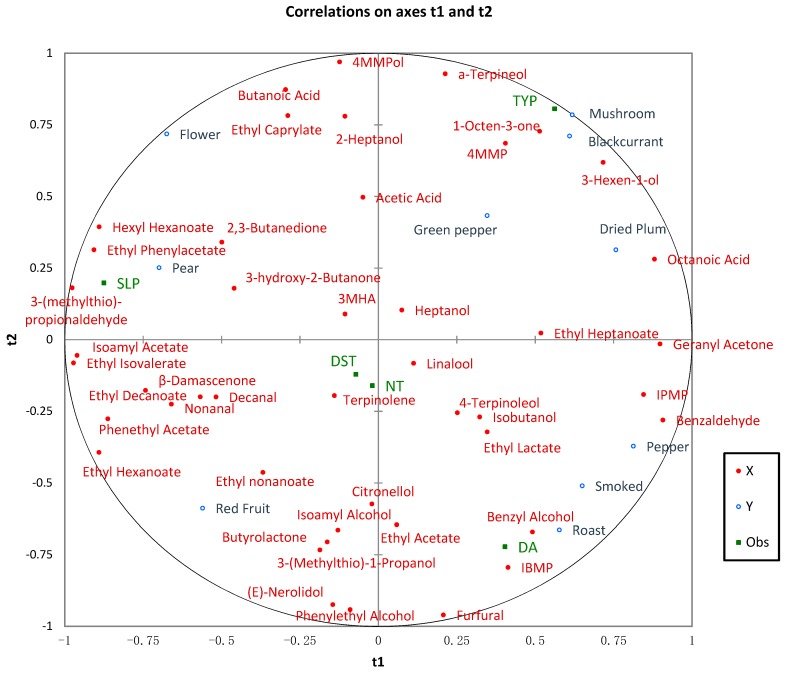
Partial least squares regression (PLSR) analysis, chemical, sensory data and the correlation of Cabernet Sauvignon wines from the Chinese Loess Plateau region (t1, sensory data; t2, chemical data). SLP, TYP, NT, DA and DST are the abbreviations for five young vineyards in the Loess Plateau region.

The PLSR has become the most popular multivariate statistical analysis method. It is a useful tool when multicollinearity exists among explanatory variables and when the number of explanatory variables is much larger than the number of observations [22]. PLSR is widely used in the research of wine, such as the relationship between sensory evaluation and flavor substances, the relevance to infrared absorption and sensory analysis, the correlation with convergence and non-volatile components, and the relations between anthocyanins and their color in aqueous solution [23,24]. Figure 2 shows the chemical and sensory results of the Cabernet Sauvignon wines overlaid over the five different vineyards in Loess Plateau, with the wines projected on to that space.

In this picture, the distance between the variable and the center of the circle shows the interpretive degree of the principal components to the variable. The greater the distance from the center point to the variable, the better the interpretation of the relations between the first two principal components and the explanatory variables. On the contrary, if the variable was near the center of the circle, it meant that much information on the variable was lost to other dimensions and the interpretive degree was low. The distance between the two variables explains the correlation between them. The closer the two variables are, the greater the positive correlation between them. If the variables are in two opposite positions away from the center of the circle, they have negative correlations. If the two variables are in the vertical position, they are not relevant.

The correlation model of the Loess Plateau Cabernet Sauvignon wine region’s sensory characteristics and aroma compounds was established to determine the influence of various aroma active substances on aroma attributes, using PLSR. As we can see from Figure 2, the aroma attributes of blackcurrant and mushroom can be used to distinguish the aroma of TYP and other regions. At the same time, TYP was also well related to 1-octen-3-one, 2-heptanol, 4MMP, 4MMPol and 3-hexen-1-ol, which showed the aroma of mushroom, blackcurrant, boxwood and green grass, respectively. SLP had high correlation with some sweet aromas such as flower and pear. It was also well related to some esters, 3-(methylthio)-propionaldehyde and 2,3-butanedione. Cabernet Sauvignon wines of DA reflected the smell of roast, smoked and pepper more. It had good relevance to benzyl alcohol, benzaldehyde, 3-isobutyl-2-methoxypyrazine (IBMP) and furfural. The red fruit was positively related to some alcohols and esters, as well as terpenes, which displayed fruity and sweet aroma. Octanoic acid was shown in close proximity to the sensory attributes of dried plum. Green pepper was near the center of the circle and IBMP and green pepper were in the vertical position that meant the explanatory relationship of IBMP to green peppers was not good. While, as reported previously, IBMP did not exhibit a direct correlation with the green pepper descriptor, it was involved in a negative relationship with the intensity rating of the bourbon character [25]. The low correlation between IBMP and green pepper character could be explained by a possible masking of IBMP by other components in the wine. PLSR applied to volatile compounds with sensory descriptor datasets showed that some descriptive sensory attributes could be well correlated to chemical profiles.

## 3. Materials and Methods

### 3.1. Chemicals

Analytical standards, with at least 97% purity were purchased from Sigma-Aldrich China Co. (St. Louis, Missouri, USA). These analytical standards were Ethyl Acetate, Ethyl Isobutyrate, Isoamyl Acetate, Ethyl Hexanoate, Ethyl Lactate, Ethyl Heptanoate, Ethyl Caprylate, Ethyl Nonanoate, Hexyl Hexanoate, Ethyl Decanoate, Isobutanol, Isoamyl Alcohol, 2-Heptanol, (E)-3-Hexen-1-ol, Heptanol, Nonanal, Decanal, 2,3-Butanedione, 3-hydroxy-2-Butanone, 1-Octen-3-one, Benzaldehyde, Benzyl Alcohol, Ethyl Phenylacetate, Phenethyl Acetate, Phenylethyl Alcohol, Terpinolene, a-Terpineol, Linalool, 4-Terpinoleol, Citronellol, Geranyl Acetone, (E)-Nerolidol, β-Damascenone, Butyrolactone, 4-Mercapto-4-methylpentan-2-one (4MMP), 3-(Methylthio)-Propionaldehyde, 4-Mercapto-4-Methyl-2-Pentanol (4MMPol), 3-(Methylthio)-1-Propanol, 3-Mercaptohexyl Acetate (3MHA), Furfural, Acetic Acid, Butanoic Acid, Octanoic Acid and O-(2,3,4,5,6-pentafluorobenzyl)hydroxylamine hydrochloride (PFBHA). L-Menthol (internal standard, IS1), Methyl Hexanoate (IS2), p-Fluorobenzaldehyde (IS3), 2,2-Dimethyl Propanoic Acid (IS4) and Isopropyl Disulfide (IS6) were purchased from ANPEL Scientific Instrument Co., Ltd. (Shanghai, China). 2-Methoxy-3-([^2^H_3_] Isobutyl) Pyrazine (IS5) was purchased from Medical Isotopes, Inc. (NH, USA). HPLC-grade ethanol absolute (≥99.8%), dichloromethane (≥99.8%) and methanol (≥99.9%) were also purchased from Sigma-Aldrich China Co. (St. Louis, MI, USA). Ultrapure water was made with Milli-Q purification system (Millipore, Bedford, MA), and it was boiled for 5 min before use.

### 3.2. Wine Samples

**Figure 3:**
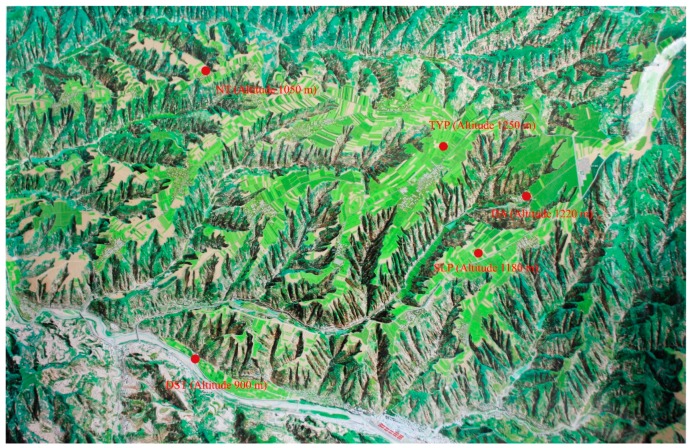
The different terrain features of five vineyards in the Chinese Loess Plateau region.

Wines from the 2015 vintage variety of Cabernet Sauvignon, taken from the following five young vineyards of the Loess Plateau region (named as SLP, TYP, NT, DA and DST) were used for this study. Figure 3 shows the terrain feature of each vineyard.

These five wines were made using the same production process. First, grapes were destemmed, crushed, and transferred to stainless steel containers after harvest. Then, SO_2_ (50–60 mg/L) and pectinase (30 mg/L) were added to the musts and the contents were mixed. After 24-h maceration of the musts, dried active yeast (200 mg/L) was added following the commercial specifications. The temperature for alcoholic fermentation condition was 28–30 °C, and the reducing sugar was less than 4 g/L. No malolactic fermentation was induced by all wine samples. After alcohol fermentation finished, the wines were chilled to −4 °C for 7 days, then 30 mg/L of free SO_2_ (on average) was added before bottling. All the samples were stored at 20 °C prior to analysis and were analyzed. Table 4 shows the basic index of five wines.

### 3.3. Panel Training

Forty subjects from Jiangnan University were recruited and trained. They all take part in wine sensory evaluation studies regularly. For descriptive analysis, 40 assessors experienced with descriptive analysis were screened based on their ability in odor identification, as well as for their cognitive (flavor memory) and descriptive language skills. Twelve assessors from 18 to 27 years old (7 males and 5 females, mean age was 22.5 years) were selected for the final test session based on their performance for providing correct answers in screening tests. Lexicons and reference standards for descriptive analysis were developed. Assessors were trained for the identification and intensive evaluation of the selected descriptors with reference standards. Panelists completed a 90-h training session in descriptive analysis and in the sensory evaluation of Cabernet Sauvignon wine. The assessors’ performance was assessed by PanelCheck (ver. 1.4.0., Nofima, Norway) in terms of their ability in consistency, stability and repeatability for giving scores before sample evaluation. The attributes and their associated reference standards are listed (Table 5).

### 3.4. Sensory Evaluation: Descriptive Analysis

All the wines were served at 20 °C in standard International Organization for Standardization (ISO) wine glasses, with watch glass lids. Double-filtered water and plain water crackers were used as palate cleansers. Subjects received 20 mL of wine for each testing. Samples were served in a randomized presentation order. Evaluations were conducted in a sensory laboratory equipped with 12 booths for individual work, under the control as required by the international standard (ISO 8589). The wine-structure descriptors were rated in the same order (0–9) for all participants. Samples were analyzed in triplicate. The subjects evaluated the samples with a 5-min break after each sample during the session. The coefficient of variance found for each subject for different replicates of each group was less than 10%.

### 3.5. Identification of Chemical Compounds by GC–O and GC–MS

A volume of 100-mL samples was added to the saturated sodium chloride, then extractions were taken three times as 20 mL, 10 mL and 10 mL volumes. Extracts were cooled to −20 °C to let the frozen water separate from the organic phase, and anhydrous sodium sulphate was added. The organic phase was concentrated to a final volume of 250 μL using a nitrogen stream.

Agilent 6890N GC coupled to an Agilent 5975 mass selective detector (MSD) and an olfactometer were used for GC–O and GC–MS analysis.

The extraction (1 μL) was injected in a splitless mode. A DB-FFAP column (60 m × 0.25 mm i.d., 0.25 μm film thickness, J&W Scientific) was used. Helium was used as a carrier gas at a constant flow rate of 2 mL/min. The injector was held at 250 °C. The column oven temperature was set at 50 °C for 2 min, then ramped to 230 °C at a rate of 6 °C/min, and held for 15 min. The column flow was split at the end of the capillary; one was directed to a heated olfactometer (Olfactory Detector Port ODP 2, Gerstel Inc., Mülheim, Ruhr, Germany), whereas the other one was directed to the MSD. The temperature of the olfactory port was 280 °C. The MS was operated in an electron impact mode (EI) at an ionization energy of 70 eV, and the ion source temperature was set at 230 °C. Full-scan acquisition was used in the 30−350 amu range of masses.

Three trained subjects from the Laboratory of Brewing Microbiology and Applied Enzymology at Jiangnan University were recruited to perform GC–O analysis. They were trained for 6 months in GC–O using 54 kinds of “Le nez du vin” (Jean Lenoir, Provence, France). The descriptors of odor were determined in the training session. During a GC run described above, subjects placed their nose close to the sniffing port, responded to the aroma intensity of the stimulus, and recorded the aroma descriptor and intensity value as well as retention time. A scale ranging from 0 to 3 (0 = none; 1 = weak, hardly recognizable note; 2 = clear but not intense note; and 3 = intense note) was used for intensity rating. The sniffing time of each run was not more than 30 min. The value for aroma intensity was averaged with the three panelists.

Each odor-active compound was identified by a comparison of mass spectra with its NIST 05 a.L database (Agilent Technologies Inc., Santa Clara, CA, USA), by comparison of its odors, retention index (RI), and mass spectra with its pure standard. The retention index of each odorant was calculated from the retention time of n-alkanes (C_5_–C_30_), according to a modified Kovats method [28].

### 3.6. Quantitative Analysis

#### 3.6.1. Headspace Solid-Phase Microextraction–Gas Chromatography–Mass Spectrometry (HS-SPME–GC–MS)

A 50/30-μm DVB/CAR/PDMS fiber (Supelco, Inc., Bellefonte, PA, USA) was used for aroma extraction. The internal standards used were: 2-Octanol (100 mg/L, IS1) and Methyl Hexanoate (91.80 mg/L, IS2). A total of 8-mL samples with a 5-μL internal standard were added into a 20-mL glass vial with a silicon septum and saturated with 3 g sodium chloride. After incubating at 60 °C for 15 min, it was extracted for 30 min under stirring at the same temperature. Then, the fiber was inserted into the injection port of GC for a 5 min desorption. The injector and oven temperatures were as the same as those used for GC–MS analysis described previously. All samples were performed in triplicate. Selective ion monitoring (SIM) mass spectrometry was used to quantitate compounds.

#### 3.6.2. HS-SPME–GC–MS after Derivatization

Quantitation of carbonyl compounds after derivatization was performed by GC−MS on a DB-FFAP column (60 m × 0.25 mm i.d., 0.25 μm film thickness, J&W Scientific) modified from literature. A sample of 8 mL saturated with NaCl was placed in a 20-mL standard headspace vial. A total of 10 μL of p-Fluorobenzaldehyde (internal standard, 100 mg/L) and 300 μL of PFBHA (20 g/L in water) were added. A 50/30-μm DVB/CAR/PDMS fiber (Supelco, Inc.,Bellefonte, PA) was used. The sample was equilibrated at 65 °C for 10 min, then extracted for 45 min under stirring at 250 rpm and desorption at 250 °C for 300 s. Sample was injected in a splitless mode. Carrier gas for the column was helium at a constant flow rate of 1 mL/min. Temperature for the injector was 280 °C. The oven temperature was 50 °C for 2 min and was increased to 100 °C at 6 °C/min for 0.1 min, then was increased to 160 °C at a rate of 2 °C/min for 0.1 min, and finally at 5 °C/min to 230 °C for 10 min. The electron impact energy was 70 eV with SIM. The ion monitored for p-Fluorobenzaldehyde after derivatization was *m*/*z* 319. The standard curve, LOD and recovery of carbonyl compounds, were measured by the method mentioned above.

#### 3.6.3. Liquid–Liquid Microextraction–Gas Chromatography–Mass Spectrometry (LLME–GC–MS)

Volatile acids were quantified by LLME–GC–MS. Due to the poor adsorption of DVB/CAR/PDMS fiber to strong polar acids, the LLME was used as a separate extraction of these acids. A total of 18 mL diluted liquor sample with 6 μL 2,2-Dimethyl Propanoic Acid (3.40 mg/L, IS4) was saturated with NaCl, then mixed for 3 min with 1 mL redistilled diethyl ether. After extraction, a 1 μL extract was injected into the injection port of the GC for analysis.

#### 3.6.4. Stable Isotope Dilution Analysis (SIDA)

The quantification of 3-Isobutyl-2-Methoxypyrazine (IBMP) and 3-Isopropyl-2-Methoxypyrazine (IPMP) was performed according to a published method [21]. In brief, 200 mL of wine (PH = 5) with dichloromethane was concentrated down to 250 μL. After extraction, 1 μ L of extract was injected into the injection port of the GC for analysis. The use of 2-Methoxy-3-([^2^H_3_] Isobutyl) Pyrazine as an internal standard (IS5) for the quantification of IBMP and IPMP. The compounds were measured using a selected ion monitoring (SIM) mode: ions *m*/*z* = 127, 154, 169 for 2-Methoxy-3-([^2^H_3_] Isobutyl) Pyrazineions. The *m*/*z* =124, 151, 169 for IBMP, *m*/*z* =137, 124 and 152 for IPMP. Ions 127, 124 and 137 were used for quantification.

#### 3.6.5. Solid-Phase Extraction–Gas Chromatography–Pulsed Flame Photometric Detector (SPE–GC–PFPD)

Methional was enriched by SPE and quantified by GC–PFPD. DB-FFAP column (30 m × 0.32 mm i.d., 1 μm film thickness, J&W Scientific Inc., Folsom, CA, USA) was used. The flow of the helium was 2 mL/min. Temperature of oven was programmed at 35 °C for 3 min and was increased to 150 °C at 10 °C/min, and held for 5 min. Then, it was increased at 20 °C/min to a final temperature of 220 °C and held for 3 min. Temperature of the GC injection and the detector was 250 °C. Sulfur gate time was 6–24.9 ms, and pulse frequency was approximately three pulses/s. Isopropyl disulfide (48.62 mg/L, 8 μL) was used as an internal standard (IS6) [12].

### 3.7. Statistical Analysis

One-way ANOVA and principal component analysis (PCA) was carried out by SPSS 19.0 (SPSS Inc., Chicago, IL, USA). Partial least squares regression (PLSR) was carried out predicting sensory descriptors (Y) with chemical concentrations (X), using the XLSTAT 2014 (Addinsoft, Paris, France).

## 4. Conclusions

This study aimed to consider and successfully demonstrate the implication of a comprehensive number of volatile compounds in and the sensory characteristics of Cabernet Sauvignon wines of the Chinese Loess Plateau region. The sensory profiles of wines form five different young vineyards in the Loess Plateau region were obtained by descriptive analysis. Blackcurrant (*p* < 0.01), pear and dried plum (*p* < 0.05), mushroom, smoked and green pepper (*p* < 0.1) had significant differences in the five vineyards. A total of 76 odor-active aroma compounds in wines were identified, and 45 odorants (Osme values ≥ 1.5) were further quantitated by five different methodologies. Multivariate statistical analysis was able to discriminate Cabernet Sauvignon wines from different vineyards (except for NT and DST), based on the concentrations of esters, alcohols, aldehyde, ketones, aromatic compounds, terpenes, sulfide, furan, acids, and pyrazines. Understanding the different sensory characteristics and the volatile composition of Cabernet Sauvignon wines from varying geographical locations of Loess Plateau has the potential to assist wine producers to brew and blend wines and establish the salient region style.

This study is beneficial to better recognizing the flavor characteristics of wines in specific regions of China. In addition, understanding the different sensory characteristics and the volatile composition of Cabernet Sauvignon wines from varying geographical locations of Loess Plateau is also conducive to establishing the unique style of wine from the region. However, the results of this study can only serve as an initial look at sensory characteristic correlations to instrumental parameters. It is, therefore, necessary to validate the reported findings on a more extensive sample set, including more vintages.

## Figures and Tables

**Table 1:** Aroma compounds identified by gas chromatography–olfactometry (GC–O) in five Cabernet Sauvignon wines.

No.	RT (min)	Aroma Compound	Odor Description	RI ^a^	Osme Values ^b^					Identification ^c^
					NT	DST	DA	SLP	TYP	
1	5.12	Ethyl Acetate	Pineapple	907	1.5	2	1.3	1.8	1.5	RI, MS, aroma
2	5.87	unknown	Fruity	-	1.8	1.3	2.4	0	1.7	aroma
3	6.38	2,3-Butanedione *	Butter	970	3	3	3	2.4	3	RI, aroma
4	6.47	Ethyl Butyrate *	Strawberry	1035	0.7	1.3	1.3	1.1	1	RI, aroma
5	6.74	2-Methyl-1-Propanol *	Banana	1053	1	1	0.8	0.8	0.5	RI, aroma
6	6.93	unknown	Gas	-	1.3	0	0	0	1.5	aroma
7	7.07	Isoamyl Acetate	Fruity	1065	1	2	1.5	1	1.2	RI, MS, aroma
8	8.19	Isobutanol	Bitter	1099	2.1	2	1	1	2.1	RI, MS, aroma
9	8.99	Isoamyl Acetate	Banana	1117	1.1	1	1.5	1.8	1.7	RI, MS, aroma
10	9.24	1-Butanol	Fruity	1140	1	0.5	0	0	1.3	RI, MS, aroma
11	9.56	Terpinolene	Lemon	1175	0.5	1.5	0.5	1	1.2	RI, MS, aroma
12	10.26	unknown	Fruity	-	1	0	0	0	2	aroma
13	10.78	unknown	Rancid, gas	-	2.3	1.5	0	0	1.5	aroma
14	11.4	3-Methyl-1-Butanol	Malt, burn	1205	2.1	3	2.5	1.5	2	RI, MS, aroma
15	11.49	unknown	Gas	-	0	2	0	0	0	aroma
16	11.69	Ethyl Hexanoate	Apple peel, fruity	1220	1	2	0	1.5	1	RI, MS, aroma
17	11.88	Styrene	Spices, gas	1241	0	0	1.1	1.1	1.3	RI, MS, aroma
18	11.96	1-Penten-3-ol	Herb	-	1	0	1.3	0	0	RI, MS, aroma
19	11.97	1-Pentanol	Fruity	1264	0	0	0	0	0.5	RI, MS, aroma
20	12.79	3-Hydroxy-2-Butanone	Butter	1304	1.5	1.7	1.1	2	1	RI, MS, aroma
21	13.25	unknown.	Nut	-	0	2	0	0	0	aroma
22	13.33	2-Heptanol	Mushroom	1303	1.8	1	1	1.5	1	RI, MS, aroma
23	13.64	1-Octen-3-one	Mushroom	1314	1.3	1.5	1	1	1	RI, MS, aroma
24	13.93	Ethyl Lactate	Fruity	1358	1.8	2.4	0.5	0	1.7	RI, MS, aroma
25	14	Ethyl Heptanoate	Fruity	1332	0	0	1.5	0	1	RI, MS, aroma
26	14.29	2-Nonanol	Cucumber	1535	0	0	0	0	0.5	RI, MS, aroma
27	16.56	a-Terpineol	Nut	1683	2	0	1.5	0	0	aroma
28	14.58	(E)-3-Hexen-1-ol	Grass	1346	1	2.4	1.1	1.2	1.5	RI, MS, aroma
29	14.62	(Z)-3-Hexen-1-ol	Pine	1361	0.5	1	0.8	0.5	0.7	RI, MS, aroma
30	14.69	4MMP *	Blackcurrant bud	1377	0	0	0	1	0	RI, MS, aroma
31	14.88	Nonanal	Fat, citrus	1385	0	2.5	1	1	2	RI, MS, aroma
32	14.92	unknown	Pine needle	1392	0	0	0.5	0	0	aroma
33	15.32	Ethyl Caprylate	Orange	1449	2.5	2.5	2	2	1.5	RI, MS, aroma
34	15.69	1-Heptanol	Herbaceous	1461	1	1	0	0	1.5	RI, MS, aroma
35	15.69	IPMP *	Green pepper	1445	1.5	1	1.1	0.5	0.5	RI, aroma
36	15.77	Acetic Acid	Vinegar	1459	1.8	1.8	1	1	1.5	RI, MS, aroma
37	15.8	1-Octen-3-ol	Mushroom	1456	0	0	0	0	1.3	RI, MS, aroma
38	16.08	3-(Methylthio)-Propionaldehyde	Cooked potato	1478	2.5	3	2.5	2	2.4	RI, MS, aroma
39	16.47	Furfural	Almond, sweet	1497	2.1	3	1.3	1.1	1.5	RI, MS, aroma
40	17.36	Ethyl 3-Hydroxybutyrate	Caramel	1490	1	1.3	0.5	0.5	1.3	RI, MS, aroma
41	17.78	Decanal	Fat, citrus	1515	0	0	0	1	2	RI, MS, aroma
42	18.08	Dipentene	Grass	-	0	1.1	0	0	0	RI, MS, aroma
43	17.6	Propanoic Acid	Spices	1527	0	0	0	0	1.3	RI, MS, aroma
44	17.67	IBMP*	Green pepper	1539	2	2	1.8	0.5	2	RI, aroma
45	17.65	Ethyl Nonanoate	Coconut	1545	0	2	0	0	1.7	RI, MS, aroma
46	17.7	2,3-Butanediol	Butter	1546	0	1	0	0	0.5	RI, MS, aroma
47	17.99	Linalool	Flowery, lavender	1537	0	0	1.5	1.5	0	RI, MS, aroma
48	18.03	4MMPol *	Grapefruit	1567	1.5	1.5	1	1	0.5	RI, aroma
49	18.16	Isobutanoic Acid	Rancid, cheese	1580	0	1.3	0	0	1	RI, MS, aroma
50	18.54	[R-(R *,R *)]-2,3-Butanediol	Fruity, lavender	1580	0	1	0	0	0	RI, MS, aroma
51	19.03	Benzaldehyde	Almond	1535	1.5	0	0	0	1.5	RI, MS, aroma
52	19.26	Hexyl Hexanoate	Fruity	1577	2	1	0	1.7	0	RI, MS, aroma
53	19.31	Terpineol	Spices	1603	0	1.5	0	0	0	RI, MS, aroma
54	19.35	Butanoic Acid	Rancid, cheese	1619	1.3	1.5	1	1	1.1	RI, MS, aroma
55	19.70	Ethyl Decanoate	Fruity	1634	0	1.7	2.4	1	1	RI, MS, aroma
56	20.18	Butyrolactone	Caramel	1641	2.1	2.4	2	2	2	RI, MS, aroma
57	20.46	Diethyl Succinate	Fruity	1683	1	1	1	0.5	1	RI, MS, aroma
58	21.28	3-(Methylthio)-1-Propanol	Cooked potato	1730	2	1	2.4	1.3	2	RI, MS, aroma
59	21.45	Methyl Salicylate	Pepper, mint	1745	1	0	1.3	1	0	RI, MS, aroma
60	21.89	Citronellol	Rose	1762	2	0	2	1.5	0	RI, MS, aroma
61	22.64	Ethyl Phenylacetate	Fruity, sweet	1772	0	1.5	1	1	0	RI, MS, aroma
62	23.02	3MHA *	Boxwood	1809	1.5	1.5	1.5	1.7	1	RI, aroma
63	23.13	Phenethyl Acetate	Honey, rose	1829	1	2	1.5	1.5	2.1	RI, MS, aroma
64	23.28	β-Damascenone *	Honey, rose	1830	3	3	3	3	3	RI, aroma
65	23.46	Hexanoic Acid	Sweat	1858	1	1.3	0	1.1	1	RI, MS, aroma
66	23.57	Ethyl Laurate	Leaf	1842	0	0	0	0.5	1	RI, MS, aroma
67	23.70	Guaiacol	Smoked	1859	0.5	1	0	0	0	RI, MS, aroma
68	23.90	Geranyl Acetone	Fruity, flowery	1872	1.5	0	0.5	0.5	1.5	RI, MS, aroma
69	24.16	Benzyl Alcohol	Sweet, flowery	1898	0.5	1	0.5	0.5	1.7	RI, MS, aroma
70	24.91	Phenylethyl Alcohol	Honey, rose	1928	1	1	1	1.8	1.5	RI, MS, aroma
71	25.22	Heptanoic Acid	Rancid, cheese	1955	0	1.3	0	0.5	0	RI, MS, aroma
72	26.50	Nerolidol	Flowery	2009	2	0	0	1.5	1.7	RI, MS, aroma
73	26.81	Diethyl Malate	Sweet	2053	1	1	0	0	1	RI, MS, aroma
74	26.98	Octanoic Acid	Sweat, cheese	2074	2.7	1	1.5	2.1	2	RI, MS, aroma
75	29.34	2-Methoxy-4-Vinylphenol	Smoked	2206	0	0	1	0	1	RI, MS
76	30.19	n-Decanoic Acid	Rancid, cheese	2269	1	0	1.3	1.3	0	RI, MS, aroma

^a^ RI = Retention index according to other literature; the meaning of “-“ was not determined; IBMP = 3-isobutyl-2-methoxypyrazine; IPMP = 3-isopropyl-2-methoxypyrazine; ^b^ Osme values, 0 = none; 1 = weak, hardly recognizable note; 2 = clear but not intense note; and 3 = intense note ^c^ Identification based on RI (retention index) or MS (mass spectrometry) or odor description.*, Confirmed by standards. SLP, TYP, NT, DA and DST are the abbreviations for five young vineyards in the Loess Plateau region.

**Table 2:** Chemical standards, quantitative ions, and calibrated intervals for Cabernet Sauvignon wines of Loess Plateau.

Compounds	CAS	Quantitative Methods	Quantitative Ion (*m*/*z*)	Slope	Intercept	*R* ^2^	Concentration Range (μg·L^−1^)	LOD (μg·L^−1^)	Recovery (%)
Esters									
Ethyl Acetate	141-78-6	HS-SPME–GC–MS	61	3.3412	1.4560	0.9972	4510.00–288,640.00	3758.33	107.03
Ethyl Isovalerate	108-64-5	HS-SPME–GC–MS	88	24.6560	0.0062	0.9967	1.45–2970.00	1.36	101.97
Isoamyl Acetate	123-92-2	HS-SPME–GC–MS	43	20.4320	−0.0304	0.9926	102.89–210,720.00	55.12	97.15
Ethyl Hexanoate	106-30-9	HS-SPME–GC–MS	88	0.5011	0.0177	0.9975	4.71–9642.60	2.52	97.03
Ethyl Lactate	97-64-3	HS-SPME–GC–MS	75	1.8127	4.5607	0.9972	965.16–988,320.00	904.84	100.57
Ethyl Heptanoate	106-30-9	HS-SPME–GC–MS	88	2.0602	−0.0004	0.9992	4.71–9642.60	4.41	115.67
Ethyl Caprylate	623-19-8	HS-SPME–GC–MS	88	11.161	−0.0563	0.9930	118.78–30,408.00	118.78	109.04
Ethyl Nonanoate	123-29-5	HS-SPME–GC–MS	88	3.6818	−0.0002	0.9959	1.33–340.32	1.05	113.23
Hexyl Hexanoate	6378-65-0	HS-SPME–GC–MS	43	0.4067	0.0078	0.9989	155.60–9958.51	137.29	108.83
Ethyl Decanoate	110-38-3	HS-SPME–GC–MS	95	3.8102	−0.0625	0.9972	38.01–19,460.00	25.63	110.78
Alcohols									
Isobutanol	78-83-1	HS-SPME–GC–MS	43	21.8640	0.0962	0.9977	80.69–41,312.00	67.24	118.34
Isoamyl Alcohol	543-49-7	HS-SPME–GC–MS	83	24.8030	0.0002	0.9996	8.43–2158.63	8.43	117.25
2-Heptanol	123-51-3	HS-SPME–GC–MS	70	1.7296	10.6250	0.9978	14871.09–3807,000.00	13121.55	98.76
(E)-3-Hexen-1-ol	928-96-1	HS-SPME–GC–MS	50	2.1288	−0.0002	0.9818	220.55–3528.80	206.77	92.32
Heptanol	111-70-6	HS-SPME–GC–MS	46	7.1471	−0.0388	0.9993	696.00–5568.00	401.54	94.99
Aldehydes									
Nonanal	124-19-6	HS-SPME–GC–MS after derivatization	83	9.6077	−0.0101	0.9910	6.73–215.38	6.31	98.12
Decanal	112-31-2	HS-SPME–GC–MS after derivatization	43	6.9755	−0.0011	0.9955	1.04–2128.32	0.98	104.03
Ketones									
2,3-Butanedione	431-03-8	HS-SPME–GC–MS after derivatization	279	5.7948	−2.6011	0.9975	3.05–24.38	2.86	92.80
3-hydroxy-2-Butanone	513-86-0	HS-SPME–GC–MS after derivatization	86	2.5245	−0.7043	0.992	7.18–1836.88	6.73	103.20
1-Octen-3-one	4312-99-6	HS-SPME–GC–MS after derivatization	140	0.7433	−0.0844	0.9971	7.18–1836.88	6.73	89.40
Aromatic compounds									
Benzaldehyde	100-52-7	HS-SPME–GC–MS	106	2.7049	−0.0193	0.9978	7.07–7240.00	5.89	95.66
Benzyl Alcohol	100-51-6	HS-SPME–GC–MS	107	4.5267	0.0303	0.9993	122.00–3904.00	101.67	97.98
Ethyl Phenylacetate	101-97-3	HS-SPME–GC–MS	129	3.0774	0.0003	0.9989	1.40–89.60	0.81	112.77
Phenethyl Acetate	103-45-7	HS-SPME–GC–MS	104	4.0966	0.0149	0.9979	10.59–5420.00	9.93	99.57
Phenylethyl Alcohol	60-12-8	HS-SPME–GC–MS	91	1.6309	0.1923	0.9992	115.28–236,100.00	96.07	101.90
Terpenes									
Terpinolene	586-62-9	HS-SPME–GC–MS	136	1.6376	0.0072	0.9866	33.32–1066.08	22.72	87.57
a-Terpineol	98-55-5	HS-SPME–GC–MS	93	8.0613	0.0010	0.983	12.31–394.00	7.10	88.48
Linalool	78-70-6	HS-SPME–GC–MS	71	21.0550	0.0016	0.999	2.14–2192.12	1.40	107.36
4-Terpinoleol	562-74-3	HS-SPME–GC–MS	136	4.3512	−0.0002	0.9976	0.42–432.90	0.35	104.30
Citronellol	106-22-9	HS-SPME–GC–MS	81	15.4540	−0.0007	0.9979	4.05–2075.20	3.20	110.11
Geranyl Acetone	3796-70-1	HS-SPME–GC–MS	320	1.6239	0.0001	0.9986	0.19–194.88	0.18	95.22
(E)-Nerolidol	40716-66-3	HS-SPME–GC–MS	64	1.7483	0.0039	0.9980	2.00–2046.00	1.07	109.63
β-Damascenone	23696-85-7	HS-SPME -GC–MS	121	4.0375	−0.0164	0.9909	3.77–241.11	2.02	119.21
Lactone									
Butyrolactone	96-48-0	HS-SPME–GC–MS	42	1.1908	0.0211	0.9981	96.00–3072.00	90.00	100.89
Sulfide									
4MMP *	19872-52-7	GC–PFPD	134	0.0117	−0.0001	0.9985	2.58–39.21	2.05	123.23
3-(Methylthio)-Propionaldehyde	3268-49-3	GC–PFPD	104	1.3862	−1.7575	0.9978	0.73–373.16	0.68	93.10
4MMPol *	31539-84-1	GC–PFPD	132	0.0505	0.0017	0.9959	10.02–380.23	8.34	85.26
3-(Methylthio)-1-Propanol	505-10-2	GC–PFPD	106	4.3283	0.0283	0.9977	1076.25–34,440.00	576.56	111.35
3MHA *	136954-20-6	GC–PFPD	116	0.1133	−0.1214	0.9994	50.23–1000.21	47.12	121.46
Furan									
Furfural	98-01-1	HS-SPME–GC–MS	39	1.3240	−0.0589	0.9905	33.70–17,256.00	25.28	102.74
Acids									
Acetic Acid	64-19-7	LLME–GC–MS	60	14.8070	7.9393	0.9996	109.44–28,016.64	98.50	120.23
Butanoic Acid	107-92-6	LLME–GC–MS	60	6.4966	−4.1557	0.9939	9.24–2365.44	7.39	109.78
Octanoic Acid	124-07-2	LLME–GC–MS	60	7.2318	−14.4220	0.9681	33.72–4316.16	30.35	99.83
Pyrazines									
IPMP *	3228-02-2	SIDA	137	7.5261	−0.6684	0.9833	1.45–2970.00	1.41	87.60
IBMP *	5508-58-7	SIDA	124	0.5490	0.0535	0.9896	10.29–21,072.00	100.67	92.50

*R*^2^: correlation coefficient. LOD: limit of detection. SIDA: stable isotope dilution analysis. “*”: ng·L^−1^. SLP, TYP, NT, DA and DST are the abbreviations for five young vineyards in the Loess Plateau region.

**Table 3:** Average values (mean ± standard deviation) of the volatile compounds in five Cabernet Sauvignon wines in Loess Plateau.

T	Compound	Content (μg/L)				
		TYP	SLP	DST	NT	DA
	Esters					
5.50	Ethyl Acetate	1670.70 ± 3.45 ^b^	2301.28 ± 4.51 ^d^	1541.92 ± 3.06 ^a^	1760.18 ± 4.64 ^c^	3247.69 ± 5.67 ^e^
7.07	Ethyl Isovalerate	5.69 ± 0.02 ^a^	34.43 ± 0.59 ^e^	16.35 ± 0.17 ^d^	14.16 ± 0.18 ^c^	12.59 ± 0.26 ^b^
9.05	Isoamyl Acetate	166.00 ± 2.51 ^a^	940.16 ± 0.14 ^e^	423.69 ± 0.95 ^d^	355.32 ± 0.22 ^c^	342.54 ± 0.19 ^b^
11.68	Ethyl Hexanoate	15.33 ± 0.55 ^a^	33.850 ± 0.62 ^c^	29.87 ± 1.08 ^c^	28.23 ± 0.95 ^d^	23.73 ± 0.73 ^b^
13.93	Ethyl Lactate	34,000.22 ± 4.15 ^d^	31,264.14 ± 1.99 ^c^	23,054.32 ± 3.54 ^b^	12,832.01 ± 4.67 ^a^	55,621.88 ± 3.63 ^e^
14.00	Ethyl Heptanoate	3.16 ± 0.23 ^c,d^	1.12 ± 0.05 ^a^	4.41 ± 0.21 ^d^	1.53 ± 0.70 ^a,b^	2.66 ± 0.37 ^b,c^
15.51	Ethyl Caprylate	4420.23 ± 3.44 ^e^	4378.07 ± 7.45 ^d^	4130.39 ± 4.75 ^c^	3107.50 ± 2.92 ^a^	3175.38 ± 8.68 ^b^
17.65	Ethyl Nonanoate	3.41 ± 1.30 ^a^	6.97 ± 0.66 ^b^	5.11 ± 0.03 ^a,b^	2.65 ± 0.24 ^a^	7.21 ± 0.09 ^b^
19.26	Hexyl Hexanoate	0.90 ± 0.01 ^b^	1.39 ± 0.02 ^e^	1.11 ± 0.01 ^d^	1.03 ± 0.01 ^c^	0.63 ± 0.01 ^a^
19.70	Ethyl Decanoate	384.84 ± 5.42 ^a^	806.360 ± 7.36 ^c^	1115.42 ± 8.91 ^d^	540.06 ± 1.30 ^b^	522.64 ± 3.30 ^b^
	Alcohols					
8.23	Isobutanol	2476.18 ± 0.28 ^d^	2354.35 ± 1.60 ^c^	1292.78 ± 5.23 ^a^	1384.78 ± 4.23 ^b^	3701.12 ± 6.12 ^e^
11.40	Isoamyl Alcohol	1677.67 ± 1.20 ^d^	1866.42 ± 3.44 ^c^	1851.10 ± 2.15 ^b^	1651.68 ± 11.60 ^d^	2010.54 ± 2.13 ^a^
13.33	2-Heptanol	50.20 ± 0.06 ^d^	42.98 ± 0.17 ^c^	29.54 ± 0.01 ^b^	48.33 ± 1.10 ^d^	23.89 ± 0.73 ^a^
14.58	(E)-3-Hexen-1-ol	1.09 ± 0.51 ^a^	0.52 ± 0.05 ^a^	0.77 ± 0.01 ^a^	0.57 ± 0.04 ^a^	0.67 ± 0.02 ^a^
16.28	Heptanol	62.57 ± 0.22b	68.13 ± 0.14c	ND	ND	76.29 ± 0.43d
	Aldehydes					
14.88	Nonanal	11.36 ± 1.27 ^a^	24.17 ± 1.99 ^c^	12.39 ± 2.24 ^a,b^	11.45 ± 0.73 ^a^	18.62 ± 1.95 ^b,c^
16.97	Decanal	7.83 ± 0.14 ^a^	19.79 ± 0.16 ^c^	6.28 ± 0.02 ^a^	6.13 ± 0.98 ^a^	15.59 ± 1.23 ^b^
	Ketones					
6.38	2,3-Butanedione	730.61 ± 0.33 ^e^	227.67 ± 0.17 ^c^	198.72 ± 0.25 ^b^	44.23 ± 0.99 ^a^	251.12 ± 1.23 ^d^
12.79	3-hydroxy-2-Butanone	483.35 ± 0.90 ^b^	529.67 ± 2.99 ^c^	578.98 ± 3.33 ^d^	602.23 ± 4.51 ^e^	411.03 ± 3.76 ^a^
13.64	1-Octen-3-one	0.08 ± 0.02 ^a^	0.09 ± 0.01 ^a^	0.08 ± 0.01 ^a^	0.08 ± 0.01 ^a^	0.08 ± 0.01 ^a^
	Aromatic compounds					
18.84	Benzaldehyde	170.89 ± 1.99 ^c^	101.30 ± 4.67 ^a^	132.20 ± 1.75 ^b^	139.71 ± 2.32 ^b^	197.35 ± 1.32 ^d^
21.19	Benzyl Alcohol	2738.97 ± 3.44 ^c^	2444.73 ± 3.50 ^a^	2471.30 ± 8.90 ^a^	2638.18 ± 12.07 ^b^	4907.43 ± 11.05 ^d^
22.86	Ethyl Phenylacetate	9.53 ± 1.87 ^b^	31.16 ± 0.96 ^c^	11.91 ± 1.23 ^b^	8.71 ± 0.34 ^a,b^	5.29 ± 0.47 ^a^
23.31	Phenethyl Acetate	38.17 ± 0.04 ^a^	78.57 ± 0.11 e	69.42 ± 0.09 ^d^	49.43 ± 0.26 ^b^	55.21 ± 0.90 ^c^
25.11	Phenylethyl Alcohol	12,543.37 ± 6.29 ^a^	16,110.19 ± 2.13 ^b^	16,156.39 ± 6.79 ^c^	18,903.91 ± 8.69 ^d^	19,803.92 ± 9.98 ^e^
	Terpenes					
9.56	Terpinolene	70.24 ± 1.64 ^c^	135.74 ± 2.67 ^d^	ND	30.46 ± 0.98 ^b^	147.74 ± 5.03 ^e^
16.47	a-Terpineol	0.05 ± 0.01 ^c^	0.02 ± 0.00 ^b^	0.01 ± 0.00 ^a,b^	ND	ND
17.79	Linalool	12.56 ± 1.87 ^b^	5.48 ± 0.43 ^a^	30.57 ± 0.68 ^d^	24.42 ± 0.83 ^c^	7.75 ± 1.01 ^a^
19.08	4-Terpinoleol	7.65 ± 0.65 ^b^	8.00 ± 0.08 ^b^	ND	0.09 ± 0.01 ^a^	15.94 ± 0.89 ^c^
22.00	Citronellol	2.30 ± 0.16 ^a^	4.59 ± 0.23 ^b^	18.54 ± 1.10 ^d^	18.31 ± 0.35 ^d^	10.05 ± 0.09 ^c^
23.70	Geranyl Acetone	10.93 ± 3.06 ^b^	2.37 ± 0.12 ^a^	3.93 ± 0.67 ^a^	5.20 ± 1.00 ^a^	11.22 ± 0.97 ^b^
26.69	(E)-Nerolidol	1.10 ± 0.19 ^a^	1.64 ± 0.16 ^a^	2.13 ± 0.12 ^a^	2.01 ± 0.90 ^a^	2.14 ± 0.33 ^a^
23.95	β-Damascenone	2.63 ± 0.17 ^a^	15.26 ± 0.74 ^c^	1.51 ± 0.34 ^a^	9.52 ± 2.90 ^b^	9.24 ± 1.34 ^b^
	Lactone					
20.08	Butyrolactone	347.12 ± 2.10 ^a^	521.92 ± 2.14 ^c^	393.94 ± 9.02 ^b^	395.24 ± 5.02 ^b^	631.16 ± 9.85 ^d^
	Sulfide					
14.69	4MMP *	3.79 ± 0.34 ^e^	1.46 ± 0.23 ^b^	1.65 ± 0.12 ^c^	3.28 ± 0.13 ^d^	1.05 ± 0.31 ^a^
16.08	3-(Methylthio)-Propionaldehyde	20.65 ± 0.67 ^a^	23.73 ± 1.31 ^b^	21.57 ± 0.84 ^a,b^	21.87 ± 0.22 ^a,b^	20.32 ± 0.46 ^a^
18.03	4MMPol *	24.21 ± 1.56 ^e^	21.60 ± 2.04 ^d^	17.56 ± 1.54 ^c^	16.50 ± 1.71 ^b^	14.26 ± 1.12 ^a^
21.15	3-(Methylthio)-1-Propanol	6294.29 ± 2.91 ^a^	9312.64 ± 2.90 ^b^	14,386.98 ± 82.00 ^e^	11,737.56 ± 74.70 ^d^	9413.23 ± 16.00 ^c^
23.02	3MHA *	266.01 ± 7.12 ^e^	253.56 ± 2.99 ^b^	255.73 ± 3.64 ^c^	519.05 ± 4.51 ^a^	171.63 ± 0.98 ^d^
	Furan					
16.43	Furfural	486.57 ± 5.00 ^a^	556.43 ± 3.60 ^b^	660.63 ± 2.10 ^d^	620.91 ± 5.18 ^c^	819.09 ± 7.67 ^e^
	Acids					
15.77	Acetic Acid	32,912.94 ± 4.701 ^e^	31,904.99 ± 9.70 ^d^	26,595.79 ± 15.00 ^b^	15,357.73 ± 4.170 ^a^	26,864.24 ± 16.49 ^c^
19.35	Butanoic Acid	1286.30 ± 8.92 ^d^	1279.59 ± 6.73 ^d^	846.21 ± 3.18 ^c^	741.70 ± 5.68 ^b^	687.95 ± 2.46 ^a^
26.98	Octanoic Acid	2183.02 ± 8.91 ^e^	806.54 ± 4.32 ^a^	949.62 ± 3.45 ^b^	1192.04 ± 2.99 ^c^	1732.84 ± 3.44 ^d^
	Pyrazines					
14.86	IPMP *	2.56 ± 0.42 ^a^	1.19 ± 0.18 ^a^	1.87 ± 0.25 ^a^	1.38 ± 0.34 ^a^	3.03 ± 0.15 ^b^
18.28	IBMP *	29.77 ± 1.52 ^a^	29.93 ± 2.15 ^a^	31.63 ± 2.56 ^a^	50.71 ± 1.71 ^b^	61.35 ± 5.11 ^c^

ND, not detected; values are means ± standard deviations. Different letters in the same row indicate that means significantly differ at *p* < 0.05. *, ng/L. SLP, TYP, NT, DA and DST are the abbreviations for five young vineyards in the Loess Plateau region.

**Table 4:** Basic information on the five different Loess Plateau region wines.

Wine Ample	pH	Total Sugar (g/L)	Total Acid (g/L)	Alcohol (%, *v*/*v*)
TYP	3.29	1.21	6.25	12.8
SLP	3.28	1.86	6.37	12.7
DST	3.32	1.45	5.82	12.4
NT	3.26	2.13	6.52	12.7
DA	3.32	1.08	5.78	13.2

**Table 5:** Cabernet Sauvignon sensory reference standards used in trained panel evaluations.

Lexicon	Aroma	Reference
1	Blackcurrant	crushed fresh or frozen blackberries [26]
2	Red berry	crushed fresh or frozen strawberry [27]
3	Smoke	4-vinylguaiacol
4	Dried plum	prune juice [26]
5	Pear	pear juice [21]
6	Chocolate	2-methoxy pyrazine
7	Mushroom	1-octen-3-ol
8	Flower	cis-rose oxide [21]
9	Green pepper	IBMP [25]
10	Pepper	black pepper corns [27]
